# Vascular Response to Graded Angiotensin II Infusion in Offspring Subjected to High-Salt Drinking Water during Pregnancy: The Effect of Blood Pressure, Heart Rate, Urine Output, Endothelial Permeability, and Gender

**DOI:** 10.1155/2014/876527

**Published:** 2014-04-17

**Authors:** Zahra Pezeshki, Fatemeh Eshraghi-Jazi, Mehdi Nematbakhsh

**Affiliations:** ^1^Water & Electrolytes Research Center, Isfahan University of Medical Sciences, Isfahan 81745, Iran; ^2^Department of Physiology, Isfahan University of Medical Sciences, Isfahan 81745, Iran; ^3^Isfahan MN Institute of Basic & Applied Sciences Research, Isfahan 81546, Iran

## Abstract

*Introduction.* Rennin-angiotensin system and salt diet play important roles in blood pressure control. We hypothesized that the high-salt intake during pregnancy influences the degree of angiotensin-dependent control of the blood pressure in adult offspring. *Methods.* Female Wistar rats in two groups (A and B) were subjected to drink tap and salt water, respectively, during pregnancy. The offspring were divided into four groups as male and female offspring from group A (groups 1 and 2) and from group B (groups 3 and 4). In anesthetized matured offspring mean arterial pressure (MAP), heart rate and urine output were measured in response to angiotensin II (AngII) (0-1000 ng/kg/min, iv) infusion. * Results.* An increase in MAP was detected in mothers with salt drinking water (*P* < 0.05). The body weight increased and kidney weight decreased significantly in male offspring from group 3 in comparison to group 1 (*P* < 0.05). MAP and urine volume in response to AngII infusion increased in group 3 (*P* < 0.05). These findings were not observed in female rats. * Conclusion.* Salt overloading during pregnancy had long-term effects on kidney weight and increased sex-dependent response to AngII infusion in offspring (adult) that may reveal the important role of diet during pregnancy in AngII receptors.

## 1. Introduction

Normal pregnancy is associated with large changes in function and anatomy of cardiovascular system. Occasionally, pregnancy is accompanied by a condition called preeclampsia, which is characterized by edema, increased intravascular coagulation, proteinuria, increased systemic vascular resistance, and hypertension. Pregnancy-induced hypertension is a major cause of maternal and fetal mortality [1–3]. High-salt intake is also often associated with increased vascular resistance and arterial pressure [3–5]. It is reported that arterial blood pressure was significantly higher in pregnant rats with high-salt intake in comparison with pregnant rats having normal-salt diet [3, 6, 7]. Maternal nutritional status during pregnancy has an important role in fetal growth [8, 9]. Furthermore, the risk of hypertension, as well as renal and cardiovascular diseases, is in part determined before birth by intrauterine factors [10]. Salt-supplemented diets cause higher sodium concentrations in the amniotic fluid in pregnant females. Lactating mothers with high-salt intake may produce milk with normal sodium concentration [7]. However, findings of previous studies indicate that the milk content in lactating mothers with high-salt intake altered [11]. Different salt diets may be recommended for blood pressure control during pregnancy as different levels of salt in utero lead to long-term consequences for health of the fetus or offspring [10]. Many systems involved in blood pressure control such as nephrogenesis, angiotensinogen expression, and renin formation are developed in early stages of pregnancy [10, 12, 13]. The salt diet may disturb these systems.

Higher blood pressure was observed in offspring from mothers subjected to high-sodium intake during pregnancy and lactation [7, 10, 14, 15]. Also, prenatally sodium-overloaded pups showed disturbances in renal development, which leads to functional and structural alterations that persist in adult life [15]. These disturbances were associated with lower plasma levels of angiotensin II (AngII), changes in renal AngII receptor type 1 (AT1R) [10], and increased urinary protein [15]. Prenatal and postnatal sodium-overloaded rats showed increased urinary protein and kidney oxidative stress, reduced glomerular filtration rate (GFR), and increased plasma volume [15].

Renin angiotensin system plays an important role in blood pressure control, and its activity was reported to be gender-related [16–21]. The main arm of RAS is angiotensin II (AngII) which plays a role in the progression of kidney disease and its activity is influenced by AT1R and AT2R [22, 23]. AT1R stimulation leads to vasoconstriction and reduction in GFR [23, 24]. In addition, in response to AngII, AT2R leads to vasodilatation [23, 25]. AT1R and AT2R expression in male and female are gender-dependent. The AT1R/AT2R ratio has also been examined in kidneys, and it is found to be lower in females because of the presence of 17*β*-estradiol [23–25].

Accordingly, it is hypothesized that different salt diets may influence renin angiotensin system or renal function in offspring. To test this hypothesis, adult male and female offspring from the mothers that received high-salt drinking water during pregnancy were subjected to AngII infusion and the blood pressure response was determined.

## 2. Methods

### 2.1. Animals

Eight female Wistar rats were individually housed in cages with temperature controlled at about 25°C and maintained with* ad libitum* standard rat diet in a 12-hour light/dark cycle. The rats were randomly divided into two groups. Rats were maintained on tap water (group A) and high-salt water (2%) (group B) before mating until delivery. High-salt water was prepared as 20 grams of salt in one liter of water. Pregnant female rats were carefully monitored at the end of pregnancy to determine the exact date of birth. On the day of weaning (30th day of life), mothers were prepared for surgery, and offspring were divided into four groups as described in the following.

 Groups 1 and 2 are male (group 1) and female (group 2) offspring from mothers who received tap water during pregnancy.
 Groups 3 and 4 are male (group 3) and female (group 4) offspring from mothers who received high-salt water during pregnancy.

All offspring in the aforementioned four groups were weighed in days 30, 45, and 60 after birth. Finally, these animals (called offspring (adult)) with the mean age of 70.2 ± 1.08 days were subjected to experimental procedures. All the animal experimental procedures described in this study were approved in advance by the Isfahan University of Medical Sciences Ethics Committee.

### 2.2. Drugs

The sodium chloride was purchased from Merck KGaA (Darmstadt, Germany); angiotensin II and Evans Blue were obtained from Sigma (St. Louis, Missouri, USA).

## 3. Experimental Protocol

### 3.1. Maternal Experimental Protocol

Thirty days after delivery, mothers in groups A and B were anesthetized with ketamine (75 mg/kg, ip), the trachea was cannulated to facilitate ventilation, and a catheter was implanted into the carotid artery. After basic stabilization for 30–60 min, blood pressure was measured and then blood sample was collected.

### 3.2. Offspring (Adult) Experimental Protocol

In experimental groups 1 to 4, the animals were weighed and anesthetized with urethane (1.5 g/kg). The trachea was isolated to insert an air ventilation tube. Then, the carotid artery was cannulated to record blood pressure and jugular vein was cannulated for AngII infusion. A catheter was implanted into the bladder to measure urine volume. Urine output was collected online during AngII infusion for period of one hour. After surgical procedures, basal blood sample (0.5 mL) was collected from the carotid artery and then centrifuged at 6000 g for 20 min to determine serum levels of nitrite (stable NO metabolite) and Na^+^; then, after the equilibrium time (30–60 min), direct blood pressure was continuously monitored. Then, a series of intravenous infusions of AngII (0, 30, 100, and 300 ng kg^−1^ min^−1^) via the jugular vein was commenced. Each dose was administered until arterial blood pressure equilibration was achieved (in about a 10 min period), and then the measurements were performed for 3–5 minutes. At the end of the study, Evans Blue [9] solution (10 mg/kg) was injected via the carotid artery, and, one hour later, all animals were sacrificed by a lethal injection of intravenous potassium chloride (10% KCL).

## 4. Measurements

The level of nitrite was measured using a colorimetric assay kit (Promega Corporation, USA) that involved the Griess reaction. Briefly, after adding sulfanilamide solution and after incubation, N-(1-naphthyl)ethylenediamine solution was added. Next, the sample absorbance was measured by a microreader in the wavelength of 492 nm. The nitrite concentration of samples was determined by comparing with the nitrite standard reference curve. The serum level of Na^+^ was also measured by a flame photometer.

### 4.1. Determination of Aortic Endothelial Permeability

Endothelial permeability of the aorta was measured by the EB method. Briefly, one hour after EB injection, a piece of thoracic aorta was obtained and immediately weighed. Then, 2 cc formamide (Merck, Germany) was added and placed in an oven (80°C) for 24 hours. After cooling, the absorbance was measured at 623 nm. The standard curve of EB concentration was plotted and the EB concentration (*μ*g) to aorta weight (gr) was determined as the endothelial permeability.

### 4.2. Serum Volume Measurement

Serum volume was measured using EB [26]. Sixty minutes after administration of EB, blood samples were collected and centrifuged. The dye concentration in the removed serum was determined at 623 nm and compared to a standard curve constructed using determined concentrations of the EB dye. The EB concentration was calculated according to the amount of EB injected to each animal and finally the serum volume was measured.

### 4.3. Statistical Analysis

Data were expressed as mean ± SEM. Body weight and MAP and heart rate (HR) response to AngII were compared via repeated measures anova for different groups of factors and doses (0, 30, 100, and 300 ng kg^−1^ min^−1^ AngII) and their interactions. The *P* value <0.05 was considered statistically significant. The Student's *t*-test was used to compare other factors between the groups. Mothers in groups A and B were compared in the number of newborn via Mann-Whitney values.

## 5. Results

All measurements were in anesthetized rats. Therefore the findings related to blood pressure may not be reflective of physiological blood pressure levels, but the conditions were similar in all groups.

### 5.1. Maternal Data

Maternal data are shown in Table 1. The data indicated that the number of male newborns in group A (mothers received tap water during pregnancy) was statistically greater than that in group B (*P* < 0.05). In group B, higher MAP and serum Na^+^ levels, and lower serum nitrite level were obtained, indicating that the salt diet during pregnancy increased MAP 30 days after delivery.

### 5.2. Offspring (Adult) Data

#### 5.2.1. MAP, HR, and Urine Volume Change in response to AngII Infusion

**Figure 1:**
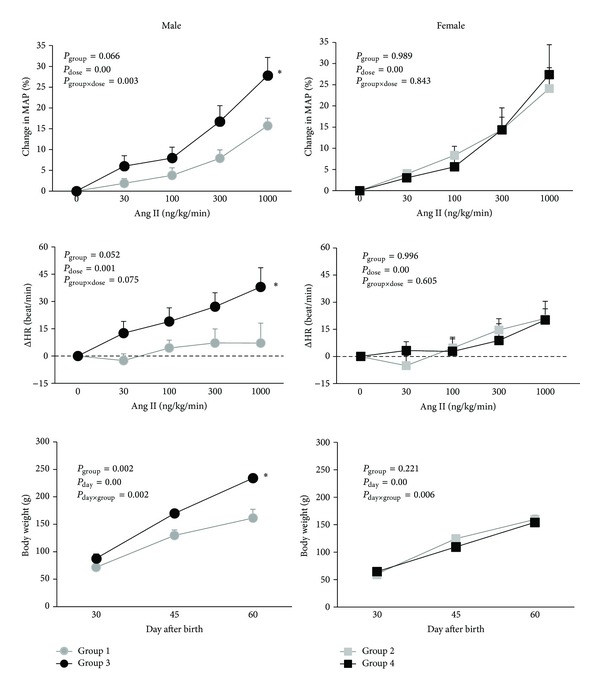
MAP and HR responses to AngII and body weight change in male (groups 1 and 3) and female (groups 2 and 4) offspring (adult). The star (∗) indicates significant difference from group 1. Groups 1 and 2: male (group 1) and female (group 2) offspring from mothers who received tap water during pregnancy. Groups 3 and 4: male (group 3) and female (group 4) offspring from mothers who received high-salt water during pregnancy.

**Figure 2:**
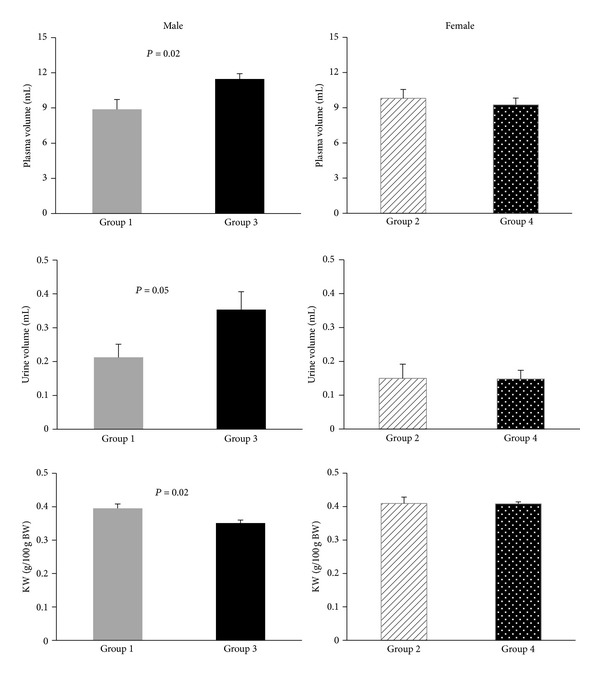
Body weight, urine output response to AngII infusion, and kidney weight in male (groups 1 and 3) and female (groups 2 and 4) offspring (adult). Groups 1 and 2: male (group 1) and female (group 2) offspring from mothers who received tap water during pregnancy. Groups 3 and 4: male (group 3) and female (group 4) offspring from mothers who received high-salt water during pregnancy.

The basal data demonstrated no significant difference between the two groups of male (groups 1 and 3) and the two groups of female animals (groups 2 and 4) (Table 2). Results in the male offspring indicate that MAP, urine volume, and HR in response to AngII were significantly higher in group 3 than those in group1 (*P* < 0.05). Such observation was not detected in female animals (Figures 1 and 2).

#### 5.2.2. Serum Levels of Nitrite and Sodium and Aorta Endothelial Permeability

Serum nitrite level in group 3 was greater than that in group 1, but the difference was not statistically significant. The groups were not significantly different with regard to the serum level of Na^+^ and endothelial permeability of aorta as EB uptake (Table 2).

#### 5.2.3. Body Weight, Kidney Weight, and Serum Volume

Changes of body weight in days 30, 45, and 60 after birth showed significant weight gain in group 3 when compared with group 1 (*P* < 0.05) (Figure 1). Also, serum volume in group 3 was higher than the volume in the other group (*P* < 0.05). However, kidney weight (KW g/100 g body weight) in group 3 was significantly less than that in group 1 (*P* < 0.05). Such finding was not observed in female groups 2 and 4 (Figure 2).

## 6. Discussion

The major findings of this study indicated that high-salt intake during pregnancy decreased kidney weight and increased plasma volume and increased the vascular, heart, and urine output responses to graded AngII infusion in male but not in female adult offspring. It is well known that salt diet is accompanied with increased vascular resistance and arterial pressure [3–5], and it increases arterial blood pressure in pregnant rats [3, 6, 7]. Higher blood pressure was observed in offspring from mothers subjected to high-sodium intake during pregnancy and lactation [7, 10, 14, 15], and certainly maternal nutritional status during pregnancy plays an important role in fetal growth [8, 9] and organ function. It seems that, among the factors that provide hypertension in offspring from mothers subjected to high-sodium intake during pregnancy, RAS is the main controlling system. Recently, Bayoglu et al. demonstrated that angiotensinogen, angiotensin converting enzyme [27], AT1R, and AT2R expression altered following maternal high-salt intake. They suggested that high-salt diet during pregnancy affects expression of the renal key elements of RAS in fetus and offspring [28]. The effect of low- and high-salt diets during pregnancy was examined on heart of male offspring, and it was concluded that high-salt diet in adult male offspring leads to higher levels of blood pressure and angiotensin II [29]. Although adaptive response to salt during pregnancy is also important for offspring [30], it is not exactly clear how other functional systems may be disturbed from this salt diet. Reduction in number of glomeruli and higher risk of hypertension may occur in offspring from mothers who were subjected to high-salt diet during pregnancy [31]. High-salt intake during pregnancy was investigated in other studies, indicating that cardiac cell and RAS may be disturbed in offspring [32, 33], but gender difference is not exactly known. Our findings also demonstrated that high-salt diet during pregnancy increased the MAP response to AngII only in male rats. However, this was not observed in female rats. It is reported that females compared with males are less sensitive to AngII [25, 34, 35]. Sensitivity to AngII is mediated by AT2R [35] and AT2R/AT1R expression ratio, which is also higher in female than male [36, 37]. It is important that when the endogenous RAS is blocked, males are still more sensitive to salt diets [38]. Accordingly, there is a possibility that high-salt diets in pregnant rats may alter the AT1R expression in male offspring but not in female. Urine volume response to AngII was also increased in male offspring from mothers who received high-salt diet, while urine volume generally decreased in these offspring [28]. The results obtained for the kidney weight in the current study were inconsistence with the results reported in other experiments [28]. Finally, significant difference was observed in serum nitrite level of mothers with salt and tap water 30 days after delivery, but no difference was observed between offspring (adults). Nitric oxide concentration is related to endothelial function [39–42]. Endothelial cells are salt sensor, and endothelial function could be changed by salt intake [43–46] because salt inactivates endothelial NO synthase in endothelial cell [47]. Therefore, the reduction of serum nitrite level in mothers with salt water is directly related to salt intake.

## 7. Conclusion

High-salt intake during pregnancy promotes MAP, heart rate, and urine output responses to AngII in male offspring. Increase in body weight and plasma volume and decrease in kidney weight were also observed in such male offspring. High-salt drinking water during pregnancy may not alter MAP, heart rate, and urine output response to AngII in female offspring. The results obtained suggest the effect of salt drinking water during pregnancy in development of RAS in male offspring.

## Figures and Tables

**Table 1:** Number of newborns, MAP, and serum nitrite level of mother with salt and tap water 30 days after delivery.

Group	Number of newborns			MAP (mmHg)	Nitrite (µmole/Lit)	Na^+^ (meq/Lit)
	Total	male	female			
A	9.7 ± 1.2	6 ± 1.5	3.7 ± 1.1	101.6 ± 2.6	35.2 ± 7.5	135.7 ± 3.9
B	6.5 ± 1.3	2.7 ± 0.2*	3.7 ± 1.0	113.8 ± 3.8*	12.3 ± 1.9*	175.7 ± 14.8*
*P* value	0.137	0.046	0.770	0.039	0.025	0.076

*Significantly different from group A.

**Table 2:** Basal values of MAP, HR, serum nitrite, Na^+^, and aorta permeability in male and female offspring (adult). Groups 1 and 2: male (group 1) and female (group 2) offspring from mothers who received tap water during pregnancy. Groups 3 and 4: male (group 3) and female (group 4) offspring from mothers who received high-salt water during pregnancy.

Gender	Group	Basal MAP (mmHg)	Basal HR (beat/min)	Basal serum nitrite (µmol/Lit)	Aorta permeability (µg/gr tissue)	Na^+^ (meq/Lit)
Male	1	115.3 ± 2.5	365 ± 14	4.71 ± 1.31	23.32 ± 4.49	132.75 ± 4.44
	3	106.2 ± 7.1	371 ± 15	11.74 ± 3.94	19.66 ± 4.35	133.5 ± 2.23
*P* value		*0.261 *	*0.775 *	*0.154 *	*0.572 *	*0.871 *
Female	2	98.1 ± 8.8	380 ± 6	8.33 ± 2.92	20.51 ± 5.09	133 ± 2.38
	4	100.9 ± 6.4	379 ± 17	5.65 ± 0.59	21.37 ± 6.20	136 ± 2.81
*P* value		*0.810 *	*0.987 *	*0.436 *	* 0.915 *	*0.457 *
